# A tribute to Lars Hennig (1970–2018)

**DOI:** 10.1093/jxb/ery337

**Published:** 2018-10-12

**Authors:** Iva Mozgova, Cristina Alexandre, Yvonne Steinbach, Maria Derkacheva, Eberhard Schäfer, Wilhelm Gruissem

**Affiliations:** 1 Institute of Microbiology, Center Algatech, Opatovický mlýn, Třeboň, Czech Republic; 2 Department of Genome Sciences, University of Washington, Seattle, Washington, USA; 3 Department of Plant and Microbial Biology, University of Zürich, Zürich, Switzerland; 4 The Sainsbury Laboratory, Norwich Research Park, Norwich, UK; 5 Institut für Biologie II, University of Freiburg, Freiburg, Germany; 7 BIOSS Centre for Biological Signalling Studies, University of Freiburg, Freiburg, Germany; 6 Department of Biology, Plant Biotechnology, ETH Zurich-LFW, Zurich, Switzerland

**Figure:**
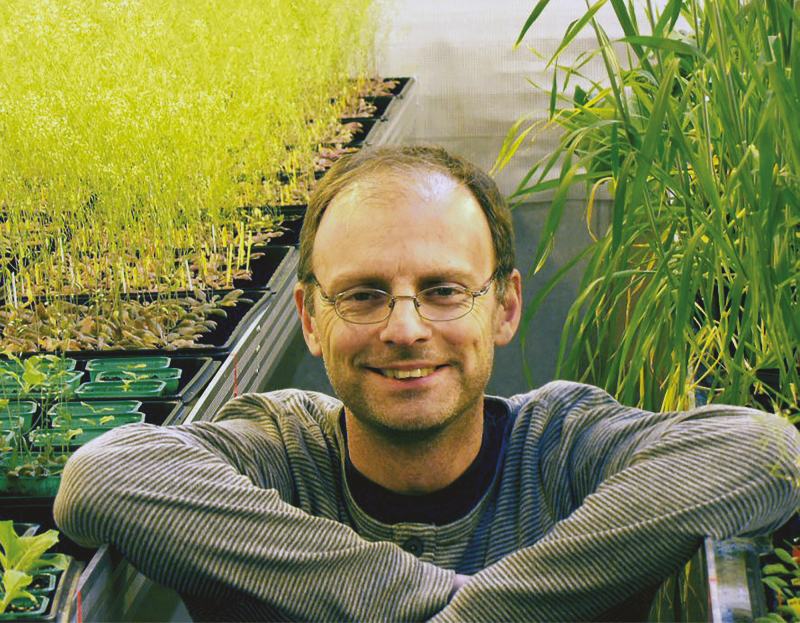
Professor Lars Hennig

Lars Hennig, Professor of Genetics at the Swedish University of Agricultural Sciences in Uppsala, Sweden, passed away on 17 May 2018, at the early age of 47. Lars was a passionate plant scientist who had a profound knowledge of biology and the determination to address fundamental questions using state-of-the-art methods. His research focused on plant developmental epigenetics, in particular the role of Polycomb group proteins and other chromatin-modifying complexes in modulating plant development and environmental responses. His extensive work is documented in over 100 scientific publications.

Lars was born in Rostock, Germany. After graduating from the Martin Luther University of Halle-Wittenberg, he moved to the Albert Ludwigs University of Freiburg in 1996. There he joined the laboratory of Eberhard Schäfer to study the dynamic behaviour and complex interactions of plant photoreceptors. Lars obtained his PhD degree in 1999. He then moved for his postdoctoral research to the ETH in Zurich where he first studied cell cycle-regulated gene expression in Wilhelm Gruissem’s laboratory. In 2003, Lars started his own research group at the ETH focusing on chromatin-based regulation of flowering time. His career as an independent researcher continued to flourish, and in 2010, he and his wife and scientific collaborator Claudia Köhler accepted full professorships at the Swedish University of Agricultural Sciences. Together with their two children they moved to Uppsala. Uprooting his research group was not without challenges, but Lars navigated the move with tact and diplomacy, from accommodating the personal circumstances of all his group members to managing the logistics of doing research during this transition period. Coming to Sweden, Lars set out to combine the best of ETH’s scientific traditions with his new cultural and scientific environment.

Although his research career was cut short by illness, Lars mentored 11 PhD students and 11 postdoctoral fellows who all successfully continued their own careers. His research led to several seminal contributions to the fields of chromatin biology and plant development.

Lars’ postdoctoral research on the different roles of MULTICOPY SUPRESSOR OF IRA 1 (MSI1) in plant development kindled his long lasting interest in chromatin dynamics and the role of chromatin-modifying complexes in regulating developmental transitions. His early work helped establish MSI1 as a subunit of two distinct chromatin-modifying complexes, CHROMATIN ASSEMBLY FACTOR 1 (CAF-1) and POLYCOMB REPRESSIVE COMPLEX 2 (PRC2). He showed that their functions were genetically separable (Hennig *et al.*, 2003; Kohler *et al.*, 2003). Later on, a significant body of work in Lars’ own group was centred on the multiple functions of MSI1, which by then he affectionately called the ‘Swiss-army-knife’. He discovered the function of MSI1-containing complexes in the control of flowering time (Bouveret *et al.*, 2006; Steinbach and Hennig, 2014), cell differentiation and reprogramming (Exner *et al.*, 2006; Mozgová *et al.*, 2017; Nakamura and Hennig, 2017), and modulation of biotic and abiotic stress responses (Alexandre *et al.*, 2009; Mehdi *et al.*, 2016; Mozgová *et al.*, 2015). Lars searched for binding partners of MSI1, and found that it linked the H3K27me3-binding LIKE HETEROCHROMATIN PROTEIN 1 (LHP1) (Turck *et al.*, 2007; Exner *et al.*, 2009) to the PRC1-PRC2 functional network. As a PRC2 component, LHP1 was proposed to be involved in the inheritance of H3K27me3 marks during cell division (Derkacheva *et al.*, 2013). LHP1 immunoprecipitation further revealed its direct interaction with PRC2 subunits, including MSI1, and identified the histone H2A deubiquitinases UBP12 and UBP13 to be physically and functionally associated with PRC2 (Derkacheva *et al.*, 2016).

Lars was enthusiastic about exploring global chromatin structure, mapping genome-wide patterns of DNA accessibility and non-canonical histone variant distribution (Shu *et al.*, 2012, 2014), developing protocols for profiling of DNA accessibility (Shu *et al.*, 2013), and identifying secondary DNA structures in intact chromatin (Gentry and Hennig, 2016). Using purified histones from cauliflower, his group identified two novel histone modifications in plants, the pericentromeric heterochromatin-associated H3K23me1 (Trejo-Arellano *et al.*, 2017) and H3K36ac associated with actively transcribed genes (Mahrez *et al.*, 2016).

While pursuing his research interests, Lars was always an active member of the plant science community. As a skilled biostatistician and bioinformatician, he and his colleagues at ETH Zurich developed pioneering functional genomic tools and established benchmarks for plant researchers. Examples include the powerful search engine Genevestigator for mining and comparative analysis of gene expression data (Zimmermann *et al.*, 2004, 2005), the AGRONOMICS1 Affymetrix microarray that expanded options for Arabidopsis transcriptomics and ChIP-chip experiments (Rehrauer *et al.*, 2010), the MIAME annotation standards for plant genome-wide profiling (Zimmermann *et al.*, 2006), and PlantDB (Exner *et al.*, 2008), a database for managing plant experiment documentation and stocks.

Together with Valérie Gaudin and Claudia Köhler, Lars initiated the successful biannual European Workshop Series in Plant Chromatin. He had an enduring fascination with the beauty and complexity of flowers. In his laboratory, flowering time reigned supreme as the developmental phenotype of choice. Outside his lab, Lars was an associate editor and the *Flowering Newsletter* editor of the *Journal of Experimental Botany* from 2012 to 2017, and established the *Flowering Highlights* blog.

Lars had an unwavering scientific curiosity, an astounding breadth of knowledge spanning different research fields, and the uncanny ability to remember seemingly all pertinent published data. As a mentor, Lars was dedicated and caring: he knew how to motivate students and postdocs at times of frustration but he also made them pause and reflect on exciting but preliminary results. His insistence on multiple experimental controls as well as the critical judgement of all data and the distinction between facts and interpretations became tenets for students and postdocs alike. Lars was committed to training the next generation of curious and rigorous scientists. He actively encouraged them to explore their career opportunities, not only by providing them with the freedom to pursue their own scientific questions but also by helping them to hone their manuscript and grant-writing skills. He wanted to see them grow as scientist and spent many hours discussing and proof-reading manuscripts.

The atmosphere around Lars was always lively and enjoyable: he liked to mingle with group members, get to know their personality and cultural background, promote discussions, and facilitate collaboration. There were laboratory lunches sweetened with Swiss chocolates, many outings, accepted manuscript celebrations, and regular after-lab beer meetings. All the BBQs, hikes in the mountains, kayaking on the Baltic Sea, and even the visit to a moose farm in the gushing rain will be fondly remembered.

We were fortunate to have worked with Lars as mentors, colleagues, collaborators, students, and postdocs. Despite his conviction that ‘life is not designed to be fair’ and his doubt about the ‘absolute truth’ in biology, Lars’ passionate quest for fairness and truth was inspiring. His sharp mind, his wisdom, his sense of humour and his friendship will be greatly missed.
